# The HAC trial (harmonic for acute cholecystitis): a randomized, double-blind, controlled trial comparing the use of harmonic scalpel to monopolar diathermy for laparoscopic cholecystectomy in cases of acute cholecystitis

**DOI:** 10.1186/1749-7922-9-53

**Published:** 2014-10-20

**Authors:** Fausto Catena, Salomone Di Saverio, Luca Ansaloni, Federico Coccolini, Massimo Sartelli, Carlo Vallicelli, Michele Cucchi, Antonio Tarasconi, Rodolfo Catena, GianLuigi De’ Angelis, Hariscine Keng Abongwa, Daniel Lazzareschi, Antonio Pinna

**Affiliations:** Department of Emergency Surgery, Parma University Hospital, Parma, Italy; St. Orsola - Malpighi University Hospital, Bologna, Italy; Bergamo Hospital, Bergamo, Italy; Macerata Hospital, Macerata, Italy

**Keywords:** Acute cholecystitis, Laparoscopic cholecistectomy, Harmonic scalpel, Biliary surgery, Safety, CBD complications, Conversion rate, Randomized controlled trial

## Abstract

**Background:**

The HARMONIC SCALPEL (H) is an advanced ultrasonic cutting and coagulating surgical device with important clinical advantages, such as: reduced ligature demand; greater precision due to minimal lateral thermal tissue damage; minimal smoke production; absence of electric corrents running through the patient. However, there are no prospective RCTs demonstrating the advantages of H compared to the conventional monopolar diathermy (MD) during laparoscopic cholecystectomy (LC) in cases of acute cholecystitis (AC).

**Methods:**

This study was a prospective, single-center, randomized trial (Trial Registration Number: NCT00746850) designed to investigate whether the use of H can reduce the incidence of intra-operative conversion during LC in cases of AC, compared to the use of MD. Patients were divided into two groups: both groups underwent early LC, within 72 hours of diagnosis, using H and MD respectively (H = experimental/study group, MD = control group). The study was designed and conducted in accordance with the regulations of Good Clinical Practice.

**Results:**

42 patients were randomly assigned the use of H (21 patients) or MD (21 patients) during LC. The two groups were comparable in terms of basic patient characteristics. Mean operating time in the H group was 101.3 minutes compared to 106.4 minutes in the control group (p=ns); overall blood loss was significantly lower in the H group. Conversion rate was 4.7% for the H group, which was significantly lower than the 33% conversion rate for the control group (p<0.05). Post-operative morbidity rates differed slightly: 19% and 23% in the H and control groups, respectively (p=ns). Average post-operative hospitalization lasted 5.2 days in the H group compared to 5.4 days in the control group (p=ns).

**Conclusions:**

The use of H appears to correlate with reduced rates of laparoscopic-open conversion. Given this evidence, H may be more suitable than MD for technically demanding cases of AC.

## Introduction

Acute cholecystitis is an acute inflammatory disease of the gallbladder. It is often attributable to gallstones, but many factors, such as ischemia, motility disorders, direct chemical injury, collagen disease, allergic reactions, and bacterial, protozoic, and parasitic infections can also play a direct role in pathogenesis [1].

Video-laparo-cholecystectomy (VLC) is a procedure used to treat patients with acute cholecystitis (AC); in 15-20% of cases, AC is the first clinical appearance of gallstones [2].

AC occurs due to persistent obstruction of the cystic duct, followed by gallbladder distension and acute inflammation. In approximately 20-40% of cases, a bacterial superinfection occurs from microorganisms of the intestinal flora [3] (Table 1).

**Table 1:** **Pathological classification of acute cholecystitis** (**according to the definitions of the Tokyo Guidelines**)

Edematous cholecystitis: first stage (***2***– ***4 days***)	***The gallbladder has interstitial fluid with dilated capillaries and lymphatics. The gallbladder wall is edematous. The gallbladder tissue is intact histologically,*** ***with edema in the subserosal layer.***
Necrotizing cholecystitis: second stage (*3*–*5 days*)	*The gallbladder has edematous changes with areas of hemorrhage and necrosis. When the gallbladder wall is subjected to elevated internal pressure*, *the blood flow is obstructed*, *with histological evidence of vascular thrombosis and occlusion. There are areas of scattered necrosis*, *but it is superficial and does not involve the full thickness of the gallbladder wall*.
Suppurative cholecystitis: third stage (*7*–*10 days*)	*The gallbladder wall has white blood cells present*, *with areas of necrosis and suppuration. In this stage*, *the active repair process of inflammation is evident. The enlarged gallbladder begins to contract and the wall is thickened due to fibrous proliferation. Intrawall abscesses are present and involve the entire thickness of the wall. Pericholecystic abscesses are present*.

Antibiotic regimens should be initiated as early as possible; these regimens should be selected and implemented according to the WSES (*World Society of Emergency Surgery*) guidelines on the treatment of intra-abdominal infections [4].

Nearly 20% of patients with AC require emergency surgery [5], and several meta-analyses have demonstrated that early VLC can decrease the length of hospital stay (LOS) and prevent disease recurrence without increasing the risk of complications [6–9].

At the outset of the laparoscopic era, VLC was thought to be contraindicated in cases of AC, and as such, it was considered “unsafe” or “technically difficult” to perform laparoscopic cholecystectomies in order to treat acute cholecystitis [10, 11].

With increasing exposure to laparoscopic surgery, a number of centers have adopted routine use of laparoscopic cholecystectomy for cases of acute cholecystitis, suggesting that it is technically feasible despite the risk of intra-operative laparoscopic-open conversion (occurring in 35% of cases [12]) and common bile duct lesions [13].

More recently, the mainstream medical community has recognized VLC as a viable treatment option for AC, as demonstrated by several prospective RCTs (*Randomized Controlled Trial*s) comparing laparoscopic and open cholecystectomies [14, 15]. It should be noted, however, that these trials reported difficulties associated with laparoscopic-open conversion.

While the surgeon’s experience plays a vital role in the treatment of acute or chronic inflammation, the employed surgical technology is also of crucial importance.

The HARMONIC SCALPEL® (H) is the leading ultrasonic cutting and coagulating surgical device, ensuring minimal charring, desiccation, and lateral thermal tissue damage.

Harmonic scalpel technology reduces ligature demand during simultaneous cutting and coagulation. Additionally, unlike conventional monopolar diathermy, patients are not exposed to electric currents to complete the device circuit, which increases the instrument’s safety. The harmonic scalpel features greater precision near vital structures and produces minimal smoke, thereby improving visibility in the immediate surgical field [16].

Several retrospective studies have demonstrated important advantages of harmonic scalpel use in laparoscopic cholecystectomies, including more effective hemostatic and biliostatic support [17–19].

However, there are currently no prospective randomized controlled trials (RCTs) investigating the advantages of the harmonic scalpel (H) compared to conventional monopolar diathermy (MD) during laparoscopic cholecystectomies (LC) in cases of acute cholecystitis (AC).

Our preliminary experience with a series of 101 cases of H-mediated laparoscopic cholecystectomy to address acute cholecystitis (AC) demonstrated a lower conversion rate (without significantly increasing morbidity rates) compared to a control group of 100 patients who underwent VLC for AC without H [20]. In this study, the mean VLC operating time for the H group was 71.4+/−14.3 minutes (range: 42–112 minutes) compared to 87.4+/−10.8 minutes for the control group (P < 0.001). Similarly, blood loss was significantly lower in the H group than it was in the MD control group. For the H group, the intra-operative conversion rate was 4.9%; the mortality rate was 1%; and the post-operative morbidity rate was 7.9%, all of which were less than or equal to the conversion, mortality, and morbidity rates of the control group: 12%, 1%, and 9%, respectively (*p* = ns).

Given this encouraging preliminary data, we designed and implemented the HAC Trial to verify these results.

This RCT was designed to test the hypothesis that, compared to conventional monopolar diathermy (MD), the harmonic scalpel (H) can effectively reduce intra-operative laparoscopic-open conversion rates for laparoscopic cholecystecomies in cases of acute cholecystitis without adversely affecting rates of post-operative morbidity.

## Materials and methods

The HAC study is a prospective, single-center, randomized controlled trial (RCT). The study was conducted in the Department of General, Emergency, and Transplant Surgery of St. Orsola-Malpighi University Hospital (Bologna, Italy) by surgeons who willingly participated in the investigation (FC, LA, SDS, ADP.).

The study abided by the guidelines of Good Clinical Practice and followed the recommendations of the Declaration of Helsinki. Additionally, the Ethics Committee of St. Orsola-Malpighi University Hospital formally approved the study on June 24, 2008. Both the study protocol and its Informed Consent documents were deemed ethically and scientifically satisfactory by the Committee, as were its objectives and general scope. Follow-up assessment of patient outcomes concluded one year after surgery.

The trial was registered with ClinicalTrials.gov and given the following identifier code: NCT00746850 [21].

Participating patients were randomly divided into two groups: in the first group, patients underwent H-mediated LC within 72 hours of diagnosis, while in the second group patients underwent MD-mediated LC within 72 hours of diagnosis. Given the conventional use of monopolar diathermy, the MD group served as the control.

Patients were randomly divided into treatment groups using computer-generated assignments. Blocked randomization was employed to ensure balance of numbers in the two groups at any given time during the study. The results of the randomized assignments were sealed in numbered envelopes, concealing the enclosed contents. If all inclusion criteria were adequately met, patients diagnosed with acute cholecystitis were asked to participate in the study. If the patient agreed, he/she would review and sign Committee-approved Informed Consent documents. Following verification of consent, the patient was randomly assigned to one of two treatment groups. The attending surgeon recorded the patient's name and treatment number [22–24]. All eligible patients were recorded [25].

### Statistical methods and power calculations

A sample size of 21 patients for each group (42 patients for the entire study) was calculated using StatCal of the Epi INFO 2000 software package (Centers for Disease Control and Prevention, Atlanta, GA, USA) to ensure a confidence level of 95% with a power > 80%.

To calculate the power a parameter with a statistically significant difference found in our preparatory retrospective study was used [20].

In particular the mean intraoperative blood loss was chosen because there was an highly statistically difference in our preparatory retrospective study and this parameter had also and high clinical importance [20].

The data were analyzed on an intention-to-treat basis. All deviations from randomized allocation, false inclusions, or missing outcomes were properly recorded.

Thorough follow-up assessment served as a safeguard measure for verifying data, as some individuals were retroactively excluded from the study based on subsequent analysis.

Missing outcomes were incorporated as baseline values, assuming the same risk as the observed participants in the control group.

The data are expressed as percentages (%) and means (±SD). The results of the compared groups were analyzed using the Pearson's Chi-Square and Fisher Exact tests, as appropriate, for proportions involving discrete data. The Fisher Exact test was used when the data were unequally distributed among the cells of the table, when the expected frequency of any cell was less than 5, or the when the total number (N) was less than 50.

For means involving continuous numerical data, the independent sample T test and the Mann–Whitney U-test were used for normally and abnormally distributed data, respectively (the data had been previously tested for normality using the Kolmogorov-Smirnov test). A *p*-value < 0.05 was considered statistically significant.

The primary outcome was based on intra-operative laparoscopic-open conversion rates. Secondary outcomes were based on intra- and post-operative morbidity and mortality rates, operating time, and intra-operative blood loss.

All secondary outcomes, including morbidity and mortality rates, operating time, and blood loss, were adjusted for qualitative imbalances (i.e. age, previous comorbidities, anticoagulant therapies, etc.).

Normal coagulation tests were determined before patients were cleared for surgery.

Inclusion criteria included the following:

- Adult patients (>18 years of age)
- Clinical (pain, fever > 37.5°C, WBC > 10.000/microL) and ultrasound evidence of cholecystitis
- ASA I-III patients
- Informed consent
- Less than 72 hours from onset of symptoms
- By contrast, exclusion criteria included the following:
- Refusal of informed consent
- Choledocholithiasis
- Generalized peritonitis
- Previous abdominal surgical procedures
- Patients with intra-operative findings indicative of non-cholecystitic pathology were retroactively excluded from the study
- Apache II score > 10

Pre-operative data included patient demographic information, comorbidity conditions (Apache II scores), and a detailed history of symptom onset.

The procedure was performed by experienced surgeons who had performed at least 50 laparoscopic cholecystectomies.

Upon admission, patients received cefotaxime, 2 grams intra-venously every 12 hours (2 g/IV/12 h), which continued post-operatively in accordance with patients’ NNISS scores.

Surgeons employed the standard four-trocars technique for laparoscopic cholecystectomies to address acute cholecystitis.

While the gallbladder was distended, its fluid content was aspirated. Large clamps were inserted through a 5 mm right lower port to achieve a secure hold on the gallbladder. In the MD (control) group, the cystic artery and duct were clip-ligated; however, in the H group, the cystic artery and duct were closed directly by H-mediated action. There was no instrumental “cross over” between the experimental (H) and control (MD) groups; for patients in the control group, surgeons only used monopolar diathermy, while in the H group, surgeons used the harmonic scalpel exclusively. The gallbladder and intra-peritoneally "dropped" stones were collected in an endoscopic retrieval bag and extracted through the umbilical port site, which was widened for removal if necessary. A closed system suction drain was placed intra-abdominally and later removed on post-operative day 1. Fascial closure was only applied at the umbilical port site, while dermal incisions at all other port sites were closed with staples. The decision to undergo intra-operative conversion to laparotomy was made by the attending surgeon, and the circumstances of each conversion were documented in detail. (Distortion of Calot’s triangle anatomy, Excessive bleeding. Time of surgery exceeding two hours).

Patients' files contained demographic information as well as pre-, peri-, and post-operative data.

Complications were classified as either surgical morbidity (wound infection, subphrenic or subhepatic abscess, bile duct injury, hemorrhage) or non-surgical morbidity (remote infections, atelectasis, deep vein thrombosis, AMI, CVA, etc.). This information was then entered into continuous or categorical variable databases, as appropriate, for further statistical analysis. Following the surgical procedure, a normal sterile dressing was applied to cover the abdomen. A second surgical team, aware of the intra-operative findings but blind to the surgical dissection instrument, then assumed care of the patient. This second team was also responsible for post-operative care and hospital discharge, evaluating the clinical outcome of patients during their recovery. During the recovery phase, the initial surgical team was always available for consultation in the event of an emergency.

Patients were deemed ready for discharge upon fulfillment of comprehensive evaluation criteria: 1) apyrexia, 2) absence of diseases requiring hospitalization, 3) return of bowel function, and 4) patient compliance. No placebo drugs were used in the course of this study. No incentives were offered to the patients to encourage surgery or follow-up assessments. The patients were free to withdraw from the study at any time.

The study’s primary objective was to demonstrate the ability of the harmonic scalpel (H) to reduce intra-operative conversion rates compared to the conventional monopolar diathermy (MD) approach during laparoscopic cholecystectomies (LC) in cases of acute cholecystits (AC); it also sought to highlight any significant differences regarding morbidity and mortality rates, operating time, and hospitalization. The primary endpoints of our study include the following:

A). To evaluate conversion rates
B). To evaluate morbidity rates, mortality rates, and operating time
C). To evaluate the duration of hospital stay

The onset of any complications were recorded at several time intervals: intra-operatively, post-operatively at time of discharge on post-operative day 7, 1 month after surgery, and 6 months after surgery.

The observed side effects did not differ significantly between the study’s control (MD) and experimental (H) groups.

Follow-up assessments were conducted 1 month, 6 months, and 1 year after surgery.

All aforementioned data were recorded in case report forms and stored in a database for the duration of the study. Statistical analysis was performed at the conclusion of the study after all particpating patients had completed the final 1-year follow-up assessment.

## Results

**Figure 1:**
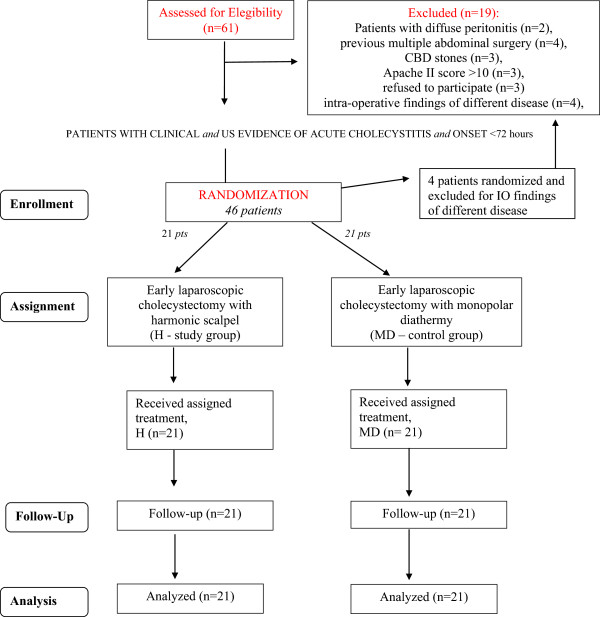
**Flow diagram of the HAC study** **(according to the Consort Statement guidelines)** **.**

**Figure 2:**
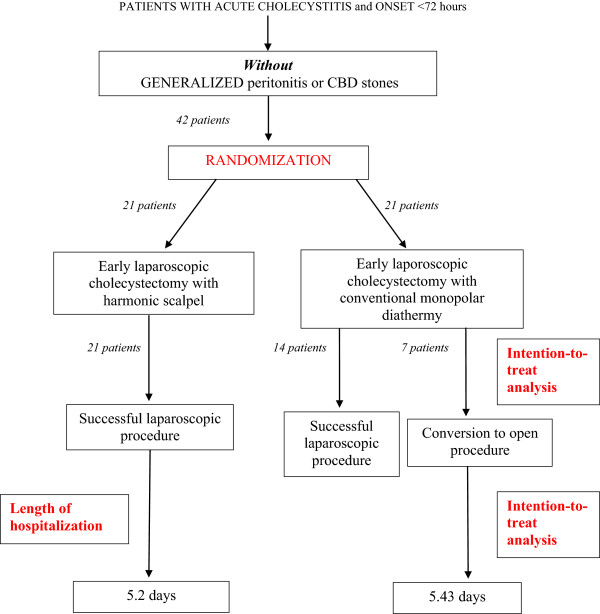
**Flow chart and results of the study.**

Of 61 potentially eligible patients, 19 were ultimately excluded: 16 subjects did not meet the inclusion criteria and 3 patients refused consent (Figure 1).42 patients were randomized to one of two treatment groups, undergoing laparoscopic cholecystectomy using either the harmonic scalpel (21 patients) or monopolar diathermy (21 patients, control group) (Figure 2).

The two groups were comparable in terms of basic patient characteristics (p = n.s.) (Table 2).

**Table 2:** **Comparison of H**-**mediated VLC for AC to VLC without the use of H for AC** (**control group**)

	VLC with H	VLC with MD	***p***
	(Study group- 21 pts)	(Control group–21 pts)	
Sex (M/F)	11/10 (52%)	10/11 (47%)	ns*
Mean age	71.2 ± 7.1	71.6 +/−6.2	ns^‡^
BMI	26.6 ± 2.1	28.1 ± 2.31	ns^‡^
Apache II score	10.1 ± 1.9	11.0 ± 2.3	ns^‡^
Time between admission and surgery (hours)	69.4 ± 6.1	68.7 ± 7.2	ns^§^
Mean operating time (minutes)	101.3 ± 10,1	106.4 ± 11.3	ns^§^
Mean intra-operative blood loss (cc)	91.1 ± 11.9	166.6 cc ± 19.2	< 0.05^§^
Conversion rate	1/21 (4.7%)	7/21 (33%)	< 0.05^†^
Morbidity rate	(5/21)23.8%	(4/21)19.0%	Ns*
Post-operative morbidity	1/21 (4.7%)	1/21 (4.7%)	ns^†^
	Conservative treatment of 1 biliary leak	Wound infection in 1 converted patient	
Non-surgical morbidity	4/21 (19%)	3/21 (14%)	ns^†^
	2 pneumonia	1 pneumonia	
	1 UTI	1 prolonged ileus	
	1 prolonged ileus	1 atrial fibrillation	
Patients with RBC transfusions	2/21 (9.5%)	1/21 (4.7%)	ns^†^
Mortality	0	0	n.a.
Post-operative hospitalization (mean)	5.2 ± 0.9	5.4 ± 1.1	ns^§^
Mean follow-up period (months)	14.2 ± 0.6	16.4 ± 0.8	ns^‡^
Late morbidity/mortality (surgery-related)	0%	0%	n.a.

Data presented as mean (SD) or number of patients (%). * = chi-square test, ^†^ = Fisher exact test, ^‡^ = independent samples T test, ^§^ = Mann–Whitney test.

The time between diagnosis and surgery was comparably long in both groups, encroaching closely on the fixed time limit of 72 hours.

Intra-operative parameters did not differ significantly between the two groups, with two notable exceptions: both blood loss (p < 0.05) and the conversion rate (p < 0.05) were found to be significantly lower for H-mediated surgery than they were for the conventional MD-mediated procedure (Table 2).

There were no major morbidities and only 2 minor surgical morbidities: 1 mild biliary leak resolved with conservative treatment (delayed abdominal drain removal after 3 days) and 1 wound infection was conservatively treated in an outpatient clinic.

Conversion rates were 4.7% and 33.4% in the H and MD (control) groups, respectively: the sole conversion to laparotomy in the H group occurred following distortion of Calot’s triangle anatomy (Table 3) whereas the MD group experienced 3 conversions due to excessive bleeding.

**Table 3:** **Overall conversion rate and reasons for conversion**

	H-mediated VLC	MD-mediated VLC
	(21 patients)	(21 patients)
Overall conversion rate	1	7
*Distortion of Calot*’*s triangle anatomy*	1	2
*Excessive bleeding*	-	3
*Time of surgery exceeding two hours*	-	2

The mortality rate was 0% for both groups, and the post-operative morbidity rates differed only slightly – 23.8% in the H group compared to 19% in the MD group – with no underlying statistical difference (Table 2).

The average length of post-operative hospital stay was 5.2 days for the H group compared to 5.4 days for the control group, again without underlying statistical difference (Table 2).

Pathology reports demonstrated a comparably high incidence of necrotizing cholecystitis in both groups without statistically significant difference (Tables 1 and 4).

**Table 4:** **Pathological classification of acute cholecystitis in the H and MD treatment groups** (**according to definitions of the Tokyo Guidelines**)

	H-mediated VLC	MD-mediated VLC	P*
	(21 patients)	(21 patients)	
Edematous cholecystitis: first stage	6 (28.5%)	5 (23.8%)	ns*
Necrotizing cholecystitis: second stage	13 (61.9%)	15 (71.4%)	ns*
Suppurative cholecystitis: third stage	2 (9.5%)	1 (4.7%)	ns*

*chi-square test.

## Discussion

Surgery is typically required in the event of acute cholecystitis due to the high risk of recurrence (30-40%) [5], and several studies have analyzed the *timing* of surgical intervention; early surgery is defined as a procedure performed within 72 hours of the onset of symptoms, while a delayed procedure is one performed 6–12 weeks after diagnosis.

The studies with the strongest evidence were reported in a series of 4 meta-analyses [6–9].

The first meta-analysis (conducted by Siddiqui [6]) reported the results of 4 RCT’s involving 375 patients; no statistically significant differences were observed in terms of the conversion rate and post-operative complications between early and delayed cholecystectomy treatment groups. Both the duration of surgery and the length of post-operative hospitalization were lower in the delayed cholecystectomy group, while the length of overall hospital stay was lower in the early cholecystectomy group.

The meta-analysis conducted by Gurusamy [7] analyzed the results of 5 RCT’s involving a total of 451 patients. No statistically significant differences were reported in the *outcomes* of the 2 groups; even the conversion rates and the incidence of iatrogenic lesions of CBD were comparable. 17.5% of the patients in the late VLC group developed recurrent cholecystitis and, among them, 40% had undergone intra-operative conversion to an *open* procedure. Additionally, post-operative hospitalization was on average 3 days shorter in the early VLC group compared to the late VLC group. However, it should be noted that the power of these studies was not strong enough to determine the rarest *outcomes*, such as those involving iatrogenic lesions.

The meta-analysis conducted by Lau [8] in 2007 included 204 patients and discovered evidence of failed non-surgical treatment in 23% of cases in the late VLC group; the study also found a statistically significant decrease in hospitalization (1.12 days) for patients in the early VLC group with no significant differences in duration of surgery, conversion rate, or morbidity rates.

The latest meta-analysis was conducted by Shikata [9] and overviewed 1,014 cases. No statistically significant differences were reported in the conversion rates between the early and late treatment groups, but post-operative hospitalization was significantly shorter for patients in the early VLC group.

As previously noted, these meta-analyses demonstrate that early VLC correlates with shorter hospitalization and reduced recurrence rates without increasing morbidity rates.

The *Tokyo Guidelines* confirmed the clinical advantages of early VLC. These guidelines also introduced a classification system of cholecystitic severity in incremental stages, highlighting the importance of draining high risk patients while allowing for delayed surgery for patients with mild, early stage, cholecystitis due to the scheduling constraints of surgical facilities [26].

These meta-analyses endorse a laparoscopic approach to treating acute cholecystitis; evidence supporting this approach is derived from 2 prospective RCTs comparing open and laparoscopic cholecystectomies.

The first of these studies (Johansson [14]) noted no difference in post-operative pain, morbidity, or recovery time. The intra-operative laparoscopic-open conversion rate was 22.8%; this metric was, by definition, only applicable to the laparoscopic treatment group. Further, the duration of surgery proved significantly longer in the laparoscopy group than in the open group (90 and 80 minutes, respectively) without differences in the median post-operative length of hospitalization (2 days in both groups).

The second study (Kiviluoto [15]) reported an intra-operative conversion rate of 16%, which was likely attributable to distortion of the cystohepatic (Calot’s) triangle. Post-operative hospital stay and recovery time were both shorter in the laparoscopic group, while post-operative complications were more prevalent in the open group; no CBD iatrogenic lesions were reported in either treatment group.

Recently, a Randomized Controlled Multicenter Trial was conducted by the Italian Polispecialistic Society of Young Surgeons (*Acute Cholecystitis Trial Invasive Versus Endoscopic Trial*; ACTIVE) [27], whose results will soon be published.

As previously noted, all of the RCTs cited in this study demonstrate high conversion rates, which have been confirmed by other retrospective studies [17, 1, 2, 9, 14].

A recent review from the UK [28] involving 43,821 laparoscopic cholecystectomies reported a 9.4% conversion rate for emergency procedures. Similarly, in a US-based study of intra-operative laparoscopic-open conversion in cases of acute cholecystitis, the national conversion rate ranged from 5% to 10% [29].

Statements by Kiviluoto [15] define the AC-driven cholecystectomy as a *technically demanding* surgical procedure requiring *experienced hands*. This issue of technical difficulty in laparoscopically treated cases of acute cholecystitis continues to strain the surgical community. Similar statements were confirmed by the Tokyo Guidelines.

In cases of acute (early VLC) or chronic inflammation (delayed VLC), even minor bleeding can obscure anatomical structures and lead to intra-operative conversion. Minor bleeding is one of the most common causes of laparoscopic-open conversion [30]. Given the demanding technical proficiency required of laparoscopic intervention, the surgeon’s prior experience greatly influences the course and outcome of the procedure.

Ultrasonically activated scalpels have proved safe and effective for laparoscopic cholecystectomies, beginning with their earliest use in 1995 [31, 32].

Several retrospective studies [17–19] have demonstrated the harmonic scalpel’s usefulness during VLC in providing hemostatic and biliostatic support, as well as addressing malacia of the cystic duct. Further, the harmonic scalpel can facilitate clip-mediated closure with reliable sealing [18]. A study by Power et al. further demonstrated the clinical efficacy of laparoscopic cholecystectomies performed with the harmonic scalpel; in a case series, duration of surgery and blood loss were both minimal, the conversion rate was low (3.9%), and no bile duct injuries occurred. The researchers concluded that use of the harmonic scalpel greatly facilitated dissection, thereby reducing operating time and decreasing the likelihood of conversion to open surgery [17]. Given its reliable technical precision in difficult cases, the harmonic scalpel also facilitates the so-called *dome*-*down cholecystectomy* [19]. Another recent study compared laparoscopic cholecystectomies performed with either the harmonic scalpel or clip and cautery; while bile leaks and bile duct-related injuries were not observed in either group, the laparoscopic group featured shorter operating time and a lower incidence of gallbladder perforation [33].

The preliminary results of our prospective series of 101 cases of acute cholecystitis treated laparoscopically using the harmonic scalpel [20] confirmed that the use of this instrument effectively reduced the intra-operative laparoscopic-open conversion rate without adversely affecting post-operative morbidity and mortality, although it should be noted that the difference in the conversion rate did not achieve statistical significance (p = 0.08). This discrepancy may be attributable to the higher incidence of second-stage necrotizing cholecystitis observed in the harmonic scalpel treatment group; such cases were included among those treated with early laparoscopic cholecystectomy given that they did not violate the 72-hour window between diagnosis and surgery. Higher incidence of necrotizing cholecystitis leads to a higher degree of anatomical distortion, thereby increasing the risk of conversion. As such, it can be inferred that an even lower conversion rate might have been observed in the H group had the treatment groups featured comparable pathology

These preliminary results were used to design the present RCT.

We hypothesize that the improved hemostatic and biliostatic support achieved using the harmonic scalpel reduces the incidence of intra-operative bleeding and biliary complications, which leads to lower rates of intra-operative conversion.

This reduction is likely more evident in technically demanding cases with a higher degree of anatomical-related difficulty, such as those involving necrotizing cholecystitis.

Our results demonstrate that patients in the H and MD groups with delayed admission to surgery featured comparable characteristics, namely a higher incidence of necrotizing cholecystitis.

Of these difficult cases, those in the harmonic scalpel group featured a lower conversion rate and a reduced mean intra-operative loss.

While there were no statistically significant differences in morbidity rates between the H and MD groups, this is likely attributable to the “protective effect” of the increased rate of intra-operative laparoscopic-open conversion in the MD group.

It should be noted, however, that the reduced conversion rate did not necessarily result in shorter post-operative hospitalization, as evidenced by 20% of non-surgical morbidities in both treatment groups involving older patients (mean age of 71 years) with severe cholecystitis (necrotizing cholecystitis in 70% of severe cases).

While instances of non-surgical morbidity influenced both treatment groups, they may have had a greater affect on the H group.

In the MD group conversion rate was 33% and this value was higher than 12% reported for MD group in the preparatory retrospective study [20].

Probably this outcome could be explained by the difference in necrotizing AC rate reported in the present RCT (70%) compared to necrotizing AC rate in the preparatory retrospective study (10%) [20].

In necrotizing AC the procedure is more difficult and the conversion rate using MD can reach 75% as reported also by other Authors [34].

Despite the low level of statistically significant difference (p = 0.04), the HAC Trial successfully demonstrated the usefulness of the harmonic scalpel in laparoscopic cholecystectomies for necrotizing cholecystitis.

## Conclusions

The use of H appears to correlate with lower intra-operative conversion rates during VLC procedures in cases of acute cholecystitis (AC) without significant increases in morbidity rates or decreases in post-operative hospitalization. The harmonic scalpel may be most useful in technically demanding cases. The use of H is recommended in cases of gangrenous AC in order to expedite the surgical procedure and reduce the likelihood of bleeding and intra-operative conversion.

To ensure cost-effectiveness, the decision to use the harmonic scalpel should be based on the results of diagnostic laparoscopy. Use of the harmonic scalpel is also recommended in cases of gangrenous cholecystitis [35].
